# Dengue 4 in Ceará, Brazil: characterisation of epidemiological and laboratorial aspects and causes of death during the first epidemic in the state

**DOI:** 10.1590/0074-02760180320

**Published:** 2018-10-18

**Authors:** Izabel Letícia Cavalcante Ramalho, Fernanda Montenegro de Carvalho Araújo, Luciano Pamplona de Góes Cavalcanti, Deborah Nunes Melo Braga, Anne Carolinne Bezerra Perdigão, Flavia Barreto dos Santos, Fernanda de Bruycker Nogueira, Kiliana Nogueira Farias da Escóssia, Maria Izabel Florindo Guedes

**Affiliations:** 1 Laboratório Central de Saúde Pública, Setor de Virologia, Fortaleza, CE, Brasil; 2 Universidade Estadual do Ceará, Rede Nordeste de Biotecnologia, Fortaleza, CE, Brasil; 3 Centro Universitário Christus, Fortaleza, CE, Brasil; 4 Universidade Federal do Ceará, Departamento de Saúde Comunitária, Fortaleza, CE, Brasil; 5 Instituto de Prevenção de Câncer, Fortaleza, CE, Brasil; 6 Instituto Oswaldo Cruz-Fiocruz, Laboratório de Imunologia Viral, Rio de Janeiro, RJ, Brasil; 7 Secretaria de Saúde do Estado do Ceará, Núcleo de Vigilância Epidemiológica, Fortaleza, CE, Brasil

**Keywords:** dengue 4, epidemiology, deaths, genotyping, Ceará/Brazil

## Abstract

BACKGROUND The first dengue cases in Brazil with laboratory confirmation occurred in the northern region of the country, with the isolation of two serotypes, dengue virus 1 (DENV-1) and DENV-4. In Ceará, the introduction of DENV-4 was reported during a DENV-1 epidemic in 2011, with only two isolations. OBJECTIVES The aim of this study was to characterise the first DENV-4 epidemic in the state of Ceará, Brazil. METHODS The study population was composed of patients with suspected dengue that were reported to health care units from January to December 2012. The laboratory confirmation of infection was made by viral isolation, reverse transcription polymerase chain reaction (RT-PCR), AgNS1, immunohistochemistry and IgM enzyme-linked immunosorbent assay (ELISA). MAIN CONCLUSIONS In the study year, 72,211 suspected dengue cases were reported and 51,865 of these cases (71.8%) were confirmed to be positive. Co-circulation of three serotypes, DENV-1, DENV-3 and DENV-4, was detected with a predominance of DENV-4 (95.3%). Most cases were not severe, but there were 44 fatal outcomes. DENV-4 Genotype II was identified for the first time in Ceará.

Dengue is one of the most serious public health problems in Brazil and in the world, especially in tropical and subtropical regions, and it affects approximately 390 million people every year, of which 96 million are clinically manifest.^1^ The disease is caused by dengue virus (DENV), an arbovirus belonging to the *Flaviviridae* family and to the *Flavivirus* genus, with four antigenically distinct serotypes, DENV-1 to 4.^2^ The four DENV serotypes exhibit high genotype variability;^3^ genomic sequencing studies have characterised five genotypes for DENV-1, six genotypes for DENV-2, five genotypes for DENV-3 and four genotypes for DENV-4.^4^

In Brazil, the first cases of dengue with laboratory confirmation occurred in Boa Vista, Roraima, in 1982, with the isolation of DENV-1 and DENV-4 serotypes.^5^ This outbreak was restricted to the northern region of the country, with no major consequences. After its introduction in Brazil, DENV-4 was isolated again in 2008 in Manaus-AM and in 2010 it was isolated in Boa Vista-RR; by the beginning of July 2011, this serotype had been isolated in 10 Brazilian states, including Ceará.^6^ ^,^ ^7^ ^,^ ^8^ In the years 2011 and 2012, DENV-4 was responsible for epidemics in most states and coincided with the simultaneous circulation of the four serotypes in the Brazilian territory. DENV-4 was first isolated in Ceará in 2011 during a DENV-1 epidemic.^9^

The aim of this study was to characterise the clinical, epidemiological and laboratorial aspects of the first DENV-4 epidemic in Ceará, Brazil.

## SUBJECTS AND METHODS

*Ethical aspects* - The study was approved by the Research Ethics Committee of the State University of Ceará, under opinion No. 913.484 and CAAE No. 38014414.6.0000.5534, and followed the Guidelines and Norms Regulating Research on Human Subjects established in Council Resolution 466/12 National Health/MS.

*Study population* - The study population was composed of patients with suspected dengue fever who reported to health units located in the 184 municipalities of the state of Ceará between January and December 2012. Data sources included the SINAN, the weekly report on dengue-SESA-CE, the Laboratory Management System (GAL) and Death Verification Service Rocha Furtado (DVS-RF) data sheets.

Case definitions

*Case of suspected dengue* - Patients with acute febrile illness, with a maximum duration of seven days, with at least two of the following symptoms: headache, retro-orbital pain, myalgia, arthralgia, prostration and rash.

*Dengue case confirmed by laboratory criteria* - A suspected case with a positive laboratory test was identified as a case of dengue confirmed by laboratory criteria.

*Dengue case confirmed by clinical-epidemiological criteria* - After the autochthonous dengue virus circulation was confirmed, acute cases of dengue were confirmed by clinical-epidemiological criteria.

*Deaths from dengue* - The criteria for suspected dengue death were based on recent reports of fever (up to seven days) without apparent bacterial infection, the presence of rash, and the presence of cavity effusion and/or bleeding.^9^ The bodies were autopsied with the consent of the family, and samples of blood, liquor and viscera were collected and sent to the laboratory to identify the cause of death.

*Laboratory diagnostic methods* - The specific laboratory diagnosis of dengue was performed in LACEN-CE through at least one of the following laboratory techniques described below:

*Viral isolation* - Viral isolation was performed by inoculation into the *Aedes albopictus* C6/36 cell line^10^ and DENV serotypes were identified by an indirect immunofluorescence (IFI) test using monoclonal antibodies specific for the four serotypes,^11^ and/or reverse transcription polymerase chain reaction (RT-PCR).^12^

*NS1 antigen capture [NS1 enzyme-linked immunosorbent assay (ELISA)]* - To capture NS1 antigen, the Platelia™ Dengue NS1 Ag-ELISA kit (Bio-Rad Laboratories, Marnes-La-Coquette, France) was used according to the manufacturer’s instructions. All samples were tested between 1-7 days after the onset of symptoms.

*IgM antibodies capture* - Anti-DENV IgM antibody capture was performed on serum samples from patients > 6 days after symptom onset using the Dengue IgM Capture ELISA kit (Panbio, Brisbane, Australia) according to the manufacturer’s instructions.

Detection of NS1 and IgM in LCR were performed as described by Araújo et al. and Soares et al.^13^ ^,^ ^14^

Molecular methods

*Viral RNA extraction* - Viral RNA was extracted using the QIAamp Viral Mini Kit (Qiagen, Inc., Valencia, USA), following the manufacturer’s instructions, and it was stored at -80ºC until RT-PCR was performed.

*RT-PCR* - Conventional RT-PCR, described by Lanciotti et al.,^12^ was used for detection and typing of DENV from acute phase samples (up to seven days of disease). The method consists of a two-step reaction, where the first step was performed by RT followed by a PCR. The protocol detects the four serotypes simultaneously in a semi-nested procedure, generating amplified products with specific sizes for each DENV serotype.

*Genotyping* - The RT-PCR technique for sequencing was performed according to the protocol described by Miagostovich et al.^15^ Oligonucleotides were used to amplify overlapping fragments of approximately 900 base pairs (bp) along the DENV-4 E gene. Amplified and purified cDNA fragments were sequenced in both directions using the BigDye Terminator Cycle Sequencing Ready Reaction kit version 3.1 (Applied Biosystems®, California, USA). After the sequencing reaction, the products were purified using Centri-Sep Spin Columns (Princeton Separation, New Jersey, USA) or a DyeEx 2.0 Spin Kit (QIAGEN, Inc., California, USA). The DNA was resuspended in 10 μL of formamide and transferred to a 96-well plate (MicroAmp Optical 96 Well Reaction Plate - Applied Biosystems, California, USA). The plate was sent to the PDTIS/Fiocruz DNA Sequencing Platform and the products were analysed by capillary electrophoresis on an ABI 3730 DNA Analyser (Applied Biosystems®, California, USA).

For analysis of the sequenced products, the program Chromas® 1.45 (http://www.technelysium.com.au/chromas14x.html) was used. Sequence identity was determined by BLAST (http://blast.ncbi.nlm.nih.gov/Blast.cgi). Sequence alignment was performed using the software ClustalW2 (http://www.ebi.ac.uk/Tools/msa/clustalw2/), and the phylogenetic tree was constructed with a substitution model based on maximum likelihood, with support of the bootstrap test (1000 pseudo-replicas) using the MEGA 5 Program (http://www.megasoftware.net/).

*Immunohistochemistry* - The immunohistochemical technique was performed on 10% formalin-fixed viscera fragments with the streptavidin-biotin-alkaline phosphatase (SAAP) technique at the Evandro Chagas Institute (IEC, Pará, Brazil). Sections of approximately 3 to 4 μm were stained with anti-DENV-2 polyclonal primary antibodies produced in mice by the IEC Arboviruses Sector. Negative and positive controls were performed for each group of samples tested.^16^

## RESULTS

In 2012, 72,211 suspected cases of dengue were reported in 184 municipalities of Ceará. Among these cases, 51,865 (71.8%) were confirmed in 168 municipalities (91.3%), with a cumulative incidence of 602.6 cases/100,000 inhabitants, Table I.

The main criterion for confirmation of cases was clinical-epidemiological (78%). These cases were concentrated in the months of April and May, reaching a peak transmission in the capital city of Fortaleza, with an incidence of 1,558.84 cases/100,000 inhabitants.

A total of 21,310 patients had blood samples collected and sent to the laboratory. The IgM ELISA test was the most widely used method in laboratory diagnosis, confirming 54% (11,511/21,310) of the cases. NS1 ELISA confirmed 17% (118/697) of the cases tested. The technique of immunohistochemistry applied to the investigation of the deaths confirmed dengue in 86.11% (31/36) of the cases analysed, Table I.

**Fig. 1:**
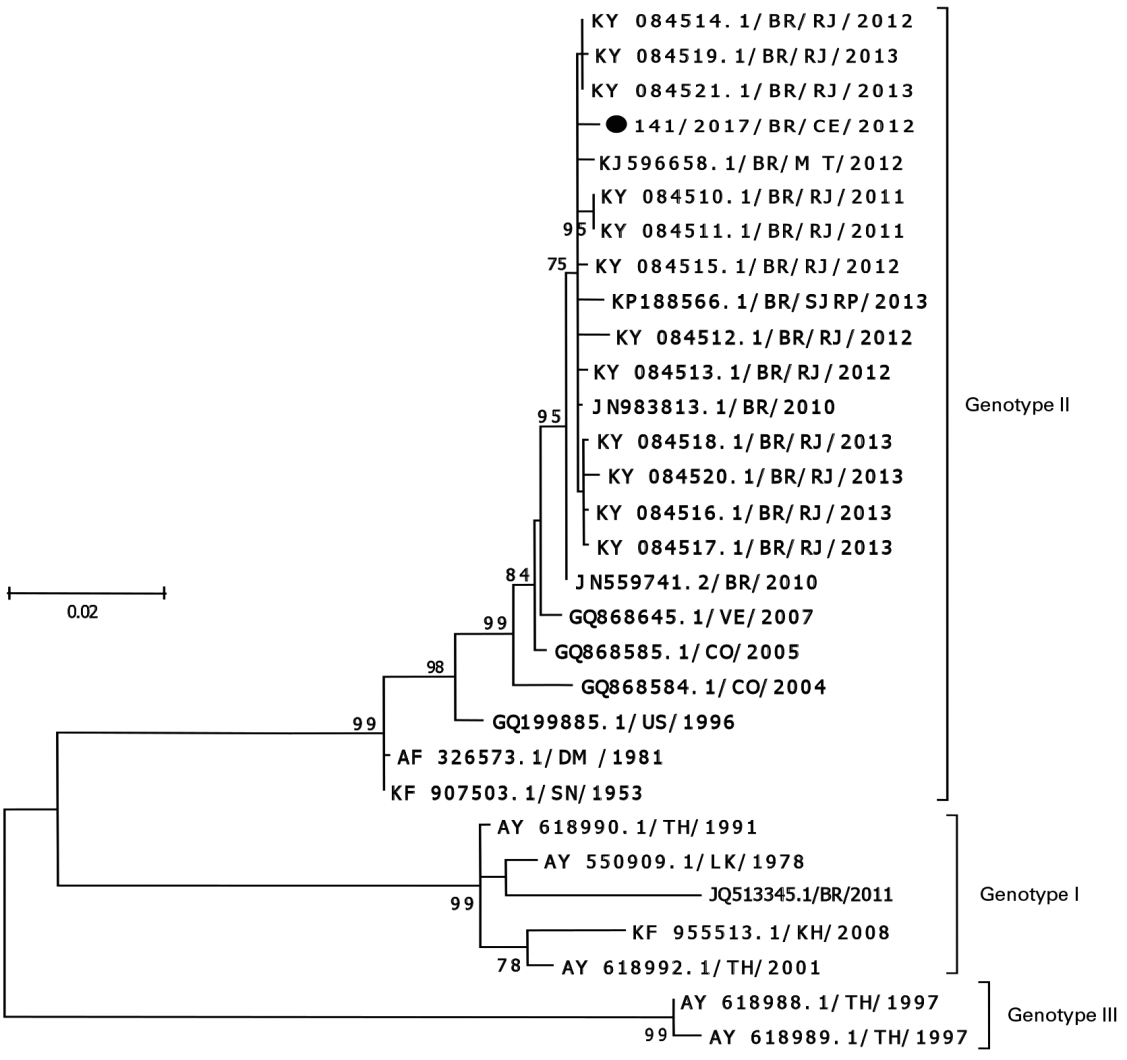
phylogenetic tree based on the dengue virus 4 (DENV-4) envelope gene from Ceará, Brazil, in 2012. The maximum-likelihood method, Tamura Nei model (TN93), discrete gamma distribution (four categories (+ G, parameter = 0.1985) of the bootstrap test (1000 pseudo-replicates) is presented at the base of the branches. The study sequence is marked with a black circle DENV reference strains were named as follows. GenBank: MH253298.

Co-circulation of three serotypes (DENV-1, DENV-3 and DENV-4), with a predominance of DENV-4 (96.3%), was detected. A representative sample of DENV-4 was analysed by viral isolation and subsequent sequencing of the E gene, allowing the identification of the circulating genotype II in the state of Ceará (Fig. 1).

**TABLE I:** Epidemiological and laboratory aspects of dengue cases occurred in Ceará, Brazil, 2012

Epidemiological aspects	(n)
Number of confirmed cases	51.865
Incidence/100,000 inhabitants	602,60
Number of municipalities	168
Diagnostic methods	Positive/Tested (%)
MAC-ELISA	11,511 / 21,310 (54)
NS1-ELISA	118/697 (17)
Viral isolation/RT-PCR (Serotypes)	108/ 975 (11) 06 DENV-1 (5.4) 01 DENV-3 (0.9) 104 DENV-4 (93.7)
Imunohistochemistry	31/36 (89)

MAC-ELISA: IgM antibody capture-enzyme-linked immunosorbent assay; RT-PCR: reverse transcription polymerase chain reaction.

**TABLE II:** Distribution of confirmed cases of dengue, by gender and age group, Ceará, Brazil, 2012

Variables	(n)	(%)
Gender		
Female	30,050	57.92
Male	21,828	42.07
Age range (years)		
< 1	613	1.18
1 a 4	1,609	3.10
5 a 9	3,005	5.79
10 a 14	5,028	9.69
15 a 19	5,943	11.46
20 a 29	12,621	24.33
30 a 39	9,318	17.96
40 a 49	6,648	12.81
50 a 59	4,110	7.92
60 a 69	1,944	3.75
70 a 79	763	1.47
≥ 80	276	0.53
Total	51,878	100.00

**TABLE III:** Classification, gender and age group of deaths confirmed by dengue, Ceará, Brazil, 2012

Variables	(nº)	(%)
Total deaths	44	100
Gender		
Male	25	57.0
Female	19	43.0
Age range (years)		
≤ 15	07	16.0
16 a 30	09	20.0
31 a 45	08	18.0
46 a 60	08	18.0
> 60	12	27.0
Total	44	100.0

**Fig. 2:**
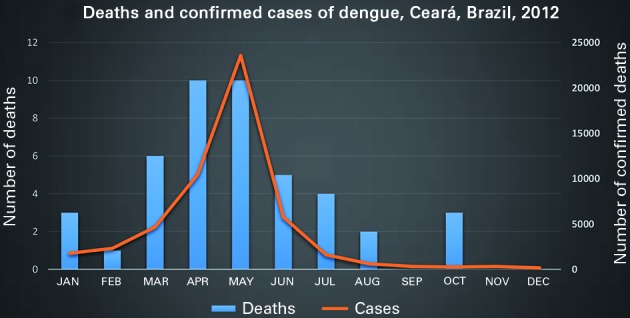
relationship between the number of confirmed cases of dengue and dengue deaths in Ceará, Brazil, 2012.

Among the cases with positive viral isolation, four cases of DENV-1 and DENV-4 co-infection were identified, and all four resulted in a cure (data not shown).

Among all the cases, predominance was observed in females (57.9%) and in the age group 20 to 29 years (24.3%), Table II.

In 2012, a total of 44 deaths from dengue were confirmed in 16 different municipalities, with a mean age of 36 years, ranging from five months to 91 years. Deaths were concentrated between March and June (Fig. 2), and 36 (82%) cadavers were autopsied. A higher percentage of deaths occurred among males (57%, 25/44) and those older than 60 years of age (Table III).

Viral isolation and/or RT-PCR identified the infectious serotype in 16 (36.4%) of the deaths, with DENV-4 being the serotype identified in 14 (87.5%) of these. Immunohistochemistry confirmed 86.2% (31/36) of the cases, Table IV.

The most frequent symptoms were fever (77.8%), vomiting (72.2%), abdominal pain (69.4%), cough (69.4%), dyspnoea (61.1%) and respiratory distress (61.1%). Comorbidities were reported in 58.3% of the deaths, primarily hypertension (30.5%), heart disease (30.5%) and diabetes mellitus (11.1%). The main associated conditions were smoking (30.5%), alcoholism (25.0%) and obesity (22.2%), Table V.

General histopathological findings such as oedema, congestion and haemorrhage predominated in the lung, brain, liver and heart. These findings also highlighted the presence of hepatitis in 80.5%, pneumonitis in 47%, myocarditis in 42% and encephalitis in 14% of the deaths that occurred in 2012, Table VI. Among the deaths, 22.7% (10/44) were positive for dengue in CSF.

## DISCUSSION

Among the confirmed cases of dengue in Ceará, regardless of the criteria of confirmation adopted, 99.6% did not progress to severe forms. In the city of Aracaju, during the epidemic in 2012, this percentage was 96.1%.^17^ Both cities exhibited percentages very close to those reported during epidemics in other Brazilian cities, where most of the symptomatic cases detected by surveillance services in Brazil progressed to less severe forms.^18^

The percentage of reported cases that were confirmed by laboratory criteria was 22%, which is considered superior to percentage of confirmations recommended by the Brazilian Ministry of Health for epidemics, which is 10% of reported cases.^19^ It should be emphasised that, for serious cases, the recommendation is always to look for laboratory techniques that confirm the diagnosis, especially for cases that progress to severe forms of disease and/or death.

The majority of the confirmed cases occurred in the population of 20 to 59 year old patients and in the female gender. This age range is clearly explained by the age distribution of the Brazilian population, but in relation to gender, it is worth noting that other analytical studies conducted in Brazil do not indicate a significant difference in the risk of illness for the female sex. There are several hypotheses to justify the higher number of female cases, especially the fact that dengue is a disease predominantly transmitted in the home and that Brazilian women are more likely than men to seek care.^17^

The confirmed cases were concentrated in the capital of Ceará, and beginning in the month of July, there was a significant reduction in the incidence, confirming the seasonality of dengue in Ceará, as well as in part of the northeast.^20^

During the 2012 epidemic, the co-circulation of three serotypes was detected, but with a marked predominance of DENV-4 (93.7%), which was similar to what occurred in other Brazilian states that same year.^21^ The predominance and dispersion of DENV-4 appeared to be predictable, since it had recently been reintroduced in Brazil, with a large part of the population still susceptible to the disease.

From the phylogenetic analysis based on the E gene, one of the samples positive for serotype 4 was characterised as belonging to genotype II, the same one that circulated in the north, northeast and southeast regions of the country in the year 2011.^22^ ^,^ ^23^ ^,^ ^24^ Genotype II, identified in our study, has been the most prevalent in the Brazilian states to date.

**TABLE IV:** Laboratory confirmation in dengue death cases (n = 44), in 2012, Ceará, Brazil

Age/G	NS1	IgM	PCR/IV	IH	Evolution (days)	Age/G	NS1	IgM	PCR/IV	IH	Evolution (days)
39/F	+	-	DENV3	-	02	76/M	-	-	DENV4	+	05
10/M	+	-	ND	-	13	27/F	-	-	-	+	10
25/F	+	-	-	-	02	13/M	-	-	DENV4	+	07
56/F	+	+	DENV1	+	05	73/M	-	-	-	+	14
62/M	+	-	ND	+	06	39/M	-	-	DENV4	+	03
41/M	+	-	DENV4	ND	02	46/F	-	-	-	+	06
52/M	+	-	DENV4	+	05	74/M	-	-	-	+	12
22/M	+	-	ND	+	03	25/F	-	-	DENV4	+	02
09/F	+	-	-	-	12	56/M	-	-	DENV4	+	03
70/M	-	+	DENV4	-	02	81/M	-	-	-	+	03
77/M	-	+	-	+	04	55/F	-	-	DENV4	+	02
91/M	-	+	ND	ND	17	56/M	-	-	DENV4	+	03
24/M	-	+	DENV4	+	07	41/F	-	-	-	+	05
5M/M	ND	+	ND	ND	10	20/M	-	-	-	+	05
39/F	-	+	ND	ND	13	04/M	-	-	DENV4	+	02
58/F	ND	+	ND	ND	05	69/M	-	-	NR	+	08
31/F	-	+	-	+	03	21/F	-	-	-	+	07
82/F	ND	+	ND	ND	-	71/M	-	-	DENV4	+	02
18/F	ND	+	ND	ND	14	65/M	-	-	-	+	03
02/F	-	+	ND	ND	05	43/F	-	-	-	+	09
53/M	-	-	-	+	05	06/F	-	-	-	+	02
25/M	-	-	DENV4	+	06	42/F	ND	ND	ND	+	03

Age/G: age/gender; F: female; M: male; NS1: NS1 ELISA; PCR/IV: polymerase chain reaction and/or viral isolation; IH: imunohistochemistry; ND: not done; (+): positive; (-) negative.

**TABLE V:** Frequency of clinical manifestation, comorbidity and risk factor reported among dengue deaths (n = 36) occurred in Ceará, Brazil

Variables	Total n (%)	Variables	Total (n)
Clinical manifestation		Comorbidity	
Fever	28 (78)	Cardiopathy	12
Vomiting	26 (72)	Hypertension	11
Cough	25 (69)	Diabetes	4
Abdominal pain	25 (69)	Hematological disease	2
Dyspnea	22 (61)	Asthma	2
Resp. distress	22 (61)	Kidney disease	2
Headache	17 (47)	Atherosclerosis	2
Irritability	16 (44)	Dyslipidemia	1
Diarrhea	15 (42)	Anemia	1
Myalgia	15 (42)	Osteoporosis	1
Prostration	14 (39)	Lung emphysem	1
Somnolence	14 (39)	Risk factor	
Hepatomegaly	13 (36)	Smoking	12
Convulsion	11 (42)	Alcoholism	9
Alt sensorium	10 (28)	Obesity	8
Exanthema	9 (25)	Sequel to stroke	2
Hematemesis	8 (22)	Transplanted	1
Arthalgia	7 (19)	Pregnancy	1
Retroorbital pain	2 (6)	Malnutrition	1

A larger number of samples should be sequenced for further investigation of the genotypic variants possibly present in the state of Ceará. Although no differences have been reported in the progression of the disease in patients with two different circulating genotypes, genotypic surveillance is necessary to identify not only the genotypes already described but also variants within the genotypes. These variants may present nucleotide alterations that may lead to changes in the viral replication process, such as changes in important amino acids in the viral structure. The genotype identification in 2012 played an important role in the molecular and genotypic surveillance of DENV-4 in the state of Ceará, since until then, there was no knowledge of the circulating genotype.

The higher incidence of male deaths can be explained by cultural influences, among other factors. There is evidence that men tend to seek health services less often than women, which can lead to a late diagnosis and thereby decrease the chances of early detection of warning signs, which increases the risk of death.^24^ One limitation of this study was that the new classification of cases proposed by the World Health Organization (WHO) was only considered in Brazil in 2014, when these data had already been recorded. Thus, we cannot consider the aspects related to the classification of cases, since there was no record of warning signs in the information system of the Ministry of Health before 2014.

**TABLE VI:** Findings of necropsies of dengue cases that evolved to death, by organ affected, Ceará, Brazil, 2012

Findings	Organ Positive/Total (%)					
	Brain	Heart	Lung	Liver	Spleen	Kidney
Oedema	35/36 (97.2)	32/36 (88.9)	36/36 (100.0)	32/36 (88.9)	31/36 (86.1)	35/36 (97,2)
Congestion	34/36 (94.4)	31/36 (86.1)	36/36 (100.0)	34/36 (94.4)	33/36 (91.7)	35/36 (97,2)
Bleeding	24/36 (66.7)	11/36 (30.5)	30/36 (83.3)	6/36 (16.7)	15/36 (41.7)	6/36 (16,7)
Necrosis	4/36 (11.1)	8/36 (22.2)	2/36 (5.5)	22/36 (61.1)	3/36 (98.3)	23/36 (63,9)
Encephalitis	5/36 (13.9)	-	-	-	-	-
Meningitis	3/36 (8.3)	-	-	-	-	-
Meningoencephalitis	2/36 (5.5)	-	-	-	-	-
Miocarditis	-	15/36 (41.6)	-	-	-	-
Fibrosis	-	12/36 (33.3)	-	-	-	-
Pericarditis	-	6/36 (16.6)	-	-	-	-
Endocarditis	-	4/36 (11.1 )	-	-	-	-
Bronchitis	-	-	26/36 (72.2)	-	-	-
Cell/typ viral change			21/36 (58.3)			
Pneumonitis	-	-	17/36 (47.2)	-	-	-
Bronchiolitis	-	-	2/36 (5.5)	-	-	-
Pleurisy	-	-	2/36 (5.5)	-	-	-
Portal infiltration	-	-	-	31/36 (86.1)	-	-
Acute hepatitis	-	-	-	29/36 (80.5)	-	-
Steatosis	-	-	-	21/36 (58.3)	-	-
Cholestasis	-	-	-	23/36 (63.8)	-	-
Councilman C	-	-	-	24/36 (66.6)	-	-
Hyd deg tub epit	-	-	-	-	28/36 (77.8)	
Monocyte infiltrate	-	-	-	-	25/36 (69.4)	-
Hyaline cylinder	-	-	-	-	22/36 (61.1)	-
Nephrosclerosis	-	-	-	-	9/36 (25.0)	-
Acute nephritis	-	-	-	-	6/36 (16.7)	-
Whi pulp hypofasia	-	-	-	-	-	26/36 (72.2)

It is also worth mentioning the high number of deaths during the 2012 dengue epidemic ranked second in Brazil in relation to the number of confirmed deaths.^18^

The CSF positivity was 20% lower than that found during the DENV-2 and DENV-3 epidemics, where the positivity reached 48,8%, suggesting less involvement of the CNS during the circulation of the DENV-4 serotype.^25^

Most autopsies revealed some type of comorbidity (61.36%), as identified in Singapore.^26^ The presence of these comorbidities, such as heart disease and hypertension, are indicated as risk factors for death.^27^

In the study period, acute pulmonary oedema was the primary immediate cause of death, reflecting the severe involvement of the respiratory system, clinically evidenced by the occurrence of respiratory distress and/or dyspnoea in 61% of the cases.

The autopsies had great relevance, considering that these studies made possible the diagnosis of dengue in 81.81% (36/44) of the deaths. The histological findings of oedema, congestion and haemorrhage in a large percentage of deaths have been described in the literature, due to haemodynamic changes associated with dengue.^28^ Histopathological changes in specific organs, such as necrosis, inflammatory processes, steatosis, degenerative changes, mainly in the liver, lung, heart and brain, have been reported in other studies.^28^

The lethality was higher than considered acceptable by the Brazilian Ministry of Health. Ceará maintains a high rate of lethality when compared to other states.^23^ This may be explained in part by the historical circulation of dengue for almost 30 years, and the existence of a local surveillance system together with a laboratory and a death verification service that identifies most of the suspected deaths.^9^

*In conclusion* - The epidemic showed the expected pattern, with most cases resulting in a cure. The laboratory confirmation of diagnosis, in detecting the new circulating serotype and confirming the emerging epidemic, was shown to be a reliable tool for disease surveillance. DENV-4 Genotype II was identified in Ceará for the first time.
